# Medicinal Plants Used by Tanzanians for Human Paediatric Ailments: A PRISMA‐Guided Systematic Review of Ethnomedicinal Evidence

**DOI:** 10.1155/tswj/1260466

**Published:** 2026-03-28

**Authors:** David Sylvester Kacholi, Neema Gideon Mogha

**Affiliations:** ^1^ Department of Biological Sciences, Dar es Salaam University College of Education, University of Dar es Salaam, P. O. Box 2329, Dar es Salaam, Tanzania, udsm.ac.tz

**Keywords:** alternative medicine, complementary medicine, ethnobotany, ethnopharmacology, herbal remedies, paediatric disease, Tanzania, traditional pharmacopoeia

## Abstract

Paediatric ailments (PAs) pose an increasing health and economic challenge in many low‐ and middle‐income countries. In Tanzania, over 80% of the rural population relies on traditional medicine for primary healthcare. Although documentation of medicinal plants (MPs) used to treat and manage PAs remains fragmented, this systematic review consolidates evidence on MPs used by Tanzanians to address these conditions. A comprehensive search was conducted across multiple databases (PubMed, Scopus, Web of Science, Google Scholar, Embase, Cochrane Library and Wiley Online Library) and grey literature sources, using predefined keywords related to PAs and MPs. The search covered publications from 1982 to 2024 and followed the Preferred Reporting Items for Systematic Reviews and Meta‐Analyses (PRISMA) guidelines. Data from 34 ethnobotanical studies were analysed using descriptive statistics in Microsoft Excel. A total of 146 MPs from 54 families were documented, with Fabaceae (10.9%) and Asteraceae (8.9%) the most frequently used families. Approximately 59.6% of MPs were collected from wild ecosystems, while 15.8% were sourced from cultivated ecosystems. The analysis of recipes shows that leaves (52.1%) and roots (39.0%) are the preferred parts, with shrubs (46.0%) the dominant life form. 63.0% of the MPs have shown positive pharmacological effects, while 37.0% lack scientific evidence but may offer potential remedies. Of the recorded MPs, 80.8% were native, and 19.2% were introduced and used to treat 51 PAs in the country. The data from this review substantially enhance the literature on MPs for paediatric diseases and can aid the development of new pharmaceutical products to reduce child mortality.

## 1. Introduction

In Tanzania, plants have historically met a wide range of essential human needs, from nutrition and shelter to medicinal uses [1]. Their role as sources of bioactive compounds has been extensively documented, with numerous species recognised for their remarkable therapeutic properties [2]. Across civilisations, medicinal plants (MPs) have been used to prevent, treat and manage diverse ailments affecting humans, underscoring their enduring relevance in healthcare systems [3, 4]. Contemporary evidence indicates that approximately 60%–80% of the Tanzanian population, particularly those residing in rural areas, relies on herbal remedies as their primary source of healthcare [5, 6]. This reliance reflects not only the accessibility of MPs but also the limited reach of conventional biomedical services, positioning ethnomedicine as a vital pillar of public health resilience [7].

Tanzania faces persistent child health challenges and remains among the 10 countries globally with the highest under‐five mortality [8]. Despite measurable progress, the mortality rate remains above the Sustainable Development Goal‐3 (SDG‐3) threshold of 12 deaths per 1000 live births. In 2023, the national under‐five mortality rate was 33 deaths per 1000 live births, a 3.36% reduction from the previous year. Nonetheless, this level remains substantially above the global target, underscoring the need for intensified interventions and sustained policy commitment [9].

Major causes of under‐five mortality include congenital heart disease, malnutrition, pneumonia, malaria, diarrhoea and anaemia [10]. Contributing systemic factors encompass inadequate rural health facilities, delayed access to care, lack of insurance coverage, poor infrastructure, insufficient staffing and recurrent shortages of essential medicines in modern health facilities [10, 11]. Consequently, many communities rely on MPs to treat and manage paediatric ailments (PAs) and associated conditions [12]. The preference for MPs is further reinforced by their accessibility, affordability and the strong local trust in traditional medicine systems [13, 14].

Despite the widespread use of MPs for PAs in Tanzania [15, 16], there is no comprehensive ethnobotanical review systematically documenting the specific plants employed for child health. Existing studies highlight their role in managing common childhood conditions, yet the evidence remains fragmented and lacks synthesis. Addressing this gap is essential for guiding pharmacological research, informing policy and strengthening paediatric healthcare strategies that integrate traditional practices with modern medicine. Therefore, this review provides a comprehensive account of scientific records on MPs employed in the management of PAs in Tanzania, while also identifying those MPs that have already undergone pharmacological investigation. This review is the first review in the country to consolidate all available literature on Tanzanian flora traditionally used in the treatment and management of PAs.

## 2. Methods

### 2.1. Search Strategy

**FIGURE 1:**
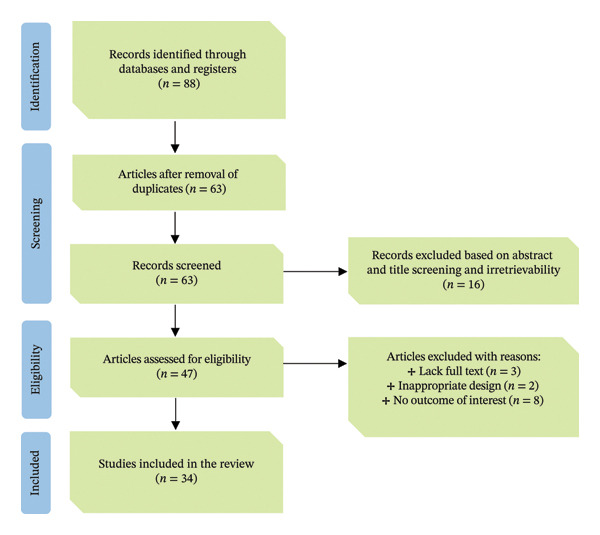
PRISMA flow diagram representing the identification and screening of the records used in the review process.

This review was conducted following the Preferred Reporting Items for Systematic Reviews and Meta‐Analyses (PRISMA) statement (Figure 1) from March to May 2025. During this period, various electronic databases, including PubMed, Scopus, Web of Science, Google Scholar, Embase, Cochrane Library, African Journals Online (AJOL) and Wiley Online Library, were searched for suitable ethnobotanical information on MPs used to treat and manage PAs in Tanzania. Additionally, information on MP used to treat and manage human PAs in the country was obtained through a systematic search of several resources not covered by electronic databases. These resources include books, book chapters, theses, dissertations, journal articles and scientific reports from the UDSM University Library. The keywords, including but not limited to, “ethnobotany,” “medicinal plants,” “phytomedicine,” “herbal remedies,” “traditional medicines,” “ethnobiology,” “ethnomedicine,” “ethnopharmacology” and “alternative medicine,” were searched in combination with other keywords such as “paediatric disorders,” “paediatric ailments,” “paediatric diseases,” “infant ailments,” “infant disorders,” “anti‐paediatric ailments or diseases or conditions,” “children’s ailments or diseases or disorders,” “United Republic of Tanzania,” and “Tanzania.” The searches were conducted independently in each database, and 34 studies (Table 1) offering valuable insights into the traditional use of MPs against PAs were included in this review. The relevant literature spanned 42 years from 1982 to 2024, a significant period that encompasses all pertinent work on the subject. The accuracy of the identified MP’s scientific names was verified using the Plants of the World Online (https://powo.science.kew.org/) botanical database.

**TABLE 1:** List of ethnobotanical studies documenting paediatric ailments in Tanzania.

Source	Region	Ethnic group	Percent
Amir et al. [17]	Mbeya, Kigoma, Shinyanga and Morogoro	Nyakyusa, Ha, Sukuma and Luguru	0.5
Amri and Kisangau [18]	Morogoro	Luguru	2.7
Augustino and Gillah [19]	Morogoro and Iringa	Luguru and Hehe	6.5
Augustino et al. [20]	Tabora	Nyamwezi	0.5
Chhabbra et al. [21]	Coast, Dar es Salaam, Tanga, Kilimanjaro and Morogoro	Ndengereko, Zaramo, Sambaa, Zigua, Chaga, Pare and Luguru	3.3
Chhabra and Mahunnah [22]	Kagera	Haya	1.6
Chhabra et al. [23]	Coast, Dar es Salaam, Tanga, Kilimanjaro and Morogoro	Ndengereko, Zaramo, Sambaa, Zigua, Chaga, Pare and Luguru	3.8
Chhabra et al. [24]	Coast, Dar es Salaam, Tanga, Kilimanjaro and Morogoro	Ndengereko, Zaramo, Sambaa, Zigua, Zigua, Chaga, Pare and Luguru	2.7
Chhabra et al. [25]	Coast, Dar es Salaam, Tanga, Kilimanjaro and Morogoro	Ndengereko, Zaramo, Sambaa, Zigua, Chaga, Pare and Luguru	1.6
Chhabra et al. [26]	Coast, Dar es Salaam, Tanga, Kilimanjaro and Morogoro	Ndengereko, Zaramo, Sambaa, Zigua, Chaga, Pare and Luguru	4.3
Chhabra et al. [27]	Coast, Dar es Salaam, Tanga, Kilimanjaro and Morogoro	Ndengereko, Zaramo, Sambaa, Zigua, Chaga, Pare and Luguru	2.2
Hedberg et al. [28]	Tanga and Kilimanjaro	Sambaa, Zigua, Chaga and Pare	6.0
Hedberg et al. [29]	Tanga and Kilimanjaro	Sambaa, Zigua, Chaga and Pare	3.8
Hedberg et al. [30]	Tanga and Kilimanjaro	Sambaa, Zigua, Chaga and Pare	4.9
Hilonga et al. [31]	Arusha, Morogoro, Mbyea, Mwanza, Dodoma	Maasai, Luguru, Nyakyusa, Sukuma and Gogo	1.1
Kacholi [32]	Morogoro	Luguru	1.6
Kacholi [33]	Morogoro	Luguru	1.6
Liheluka et al. [15]	Tanga	Sambaa and Zigua	26.1
Liheluka et al. [34]	Tanga	Sambaa, Bondei and Zigua	2.2
Maregesi et al. [35]	Mara	Kurya and Zanaki	1.1
Mollel et al. [5]	Arusha	Maasai	1.1
Moshi et al. [36]	Kagera	Haya	2.2
Moshi et al. [37]	Kagera	Haya	1.6
Moshi et al. [38]	Kagera	Haya	6.5
Nahashon [39]	Dar es Salaam	Zaramo	3.3
Nondo et al. [40]	Kagera and Lindi	Haya and Makuwa	0.5
Posthouwer [41]	Dar es Salaam	Zaramo	0.5
Qwarse et al. [42]	Manyara	Barbeig	0.5
Ranathal and Ngassapa [43]	Kagera	Tutsi	1.1
Ruffo [44]	Tabora	Nyamwezi	1.1
Said and Peter [45]	Tanga	Sambaa	0.5
Shangali et al. [46]	Iringa	Hehe	1.1
Shija et al. [47]	Coast	Zaramo and Ndengereko	0.5
Temu et al. [48]	Manyara	Barbaig	1.1

### 2.2. Inclusion Criteria

The review was restricted to original ethnobotanical investigations conducted in Tanzania that reported paediatric information. Studies were eligible if published or unpublished, provided they were available in English. Inclusion was contingent upon the documentation of the botanical name, local name, plant parts utilised, methods of preparation and routes of administration.

### 2.3. Exclusion Criteria

Studies were excluded if they were review articles, letters or other publications that could not be retrieved. Research lacking sufficient MP data, such as missing scientific names, plant parts used, preparation methods, routes of administration or study locations, was also omitted. Additionally, studies that were methodologically weak, restricted to MPs for livestock, conducted outside Tanzania, or published in languages other than English were excluded from the review.

### 2.4. Data Analysis

Ethnobotanical data were processed and analysed in Microsoft Excel to generate descriptive statistics on MPs, families, utilised plant parts and growth forms. Results were presented in tabular and graphical formats.

## 3. Results and Discussion

### 3.1. Sources and Distribution of the MPs in the Country

This review documents 34 ethnobotanical surveys offering MP from 17 of the 31 administrative regions in the country (Table 1). The results indicate that there are limited studies on the prevalence of PAs in the country. Most reviewed studies have been conducted in the Tanga, Morogoro, Kilimanjaro, Dar es Salaam, Coast and Kagera regions (Table 1), with many MPs being recorded in these regions. This high number suggests that locals in these areas possess significant traditional knowledge of MPs for PAs; it is also possible that these regions experience a higher frequency of PAs. Other reviewed studies were from Tabora, Shinyanga, Mwanza, Kigoma, Mbeya, Dodoma, Arusha, Mara, Manyara, Lindi and Iringa (Table 1).

### 3.2. MP Diversity

A total of 146 MPs from 54 families were identified by Tanzanians as being used to treat and manage PAs. The most frequently used plants belong to the family Fabaceae (16 species, 10.9%), followed by Asteraceae (13 species, 8.9%), Rubiaceae (9 species, 6.2%), Lamiaceae (8 species, 5.5%) and Solanaceae, Rutaceae and Malvaceae (5 species each, 3.4%) (Table 2). The predominant use of these families for managing PAs and related complications is attributed to their wide range of bioactive compounds, which make them particularly effective in addressing PAs and other ailments [13]. Similar to the findings of this study, research conducted in South Africa [49], Zimbabwe [50] and Uganda [13] has also reported Fabaceae, Asteraceae and Lamiaceae as frequently used botanical families in managing PAs. As in many African countries, Tanzanians typically use MPs to treat and manage PAs and other ailments due to their affordability, cultural acceptability, ease of access and fewer side effects [1, 51, 52]. This suggests that MPs are essential alternatives to conventional PHA remedies, particularly for low‐income families. Moreover, the widespread and high utilisation of MPs from the aforementioned families indicates their broad distribution in Africa and worldwide.

**TABLE 2:** Medicinal plants used for treating paediatric ailments in Tanzania.

Family	Species name	Local name	OG	So	LF	PU	CS	Ailments (reference)
Acanthaceae	*Justicia engleriana* Lindau	Ekishenda (Hay)	N	W	S	L, R	—	Coughs [38]
	*Justicia striata* (Klotzsch) Bullock	Akalaza (Hay)	N	W	H	L	—	Antipyretic [36]
	*Whitfieldia elongata* (P.Beauv.) De Wild. & T. Durand	Ekigenge/Lugenge (Hay)	N	C, W	S	Ap	—	Chicken pox and skin conditions [36, 38]

Aeschynomene	*Aeschynomene elaphroxylon* (Guill. & Perr.) Taub.	Makubeshta (Bar)	N	C, W	S	L	LC	Intestinal worms [48]

Amaranthaceae	*Achyranthes aspera* L.	Sitahula (Sa), Mzangaze (Sw)	I	C	H	L	—	Diarrhoea [15]
	*Chenopodium opulifolium* Schrad. ex W.D.J.Koch & Ziz	Mfunguo (Sw)	N	W	H	L, B	—	Asthma and flu [39]

Amaryllidaceae	*Allium gomphrenoides* Boiss. & Heldr.	Kitunguu saumu (Sw)	I	C	B	R, L	DD	Fever [18]
	*Allium sativum* L.	Kitunguu maji (Sw)	I	C	B	L	—	Fever [18]
	*Crinum papillosum* Nordal	Mfunguo (Sw)	N	C, W	B	B, L		Asthma and flu [39]

Anacardiaceae	*Lannea schweinfurthii* (Engl.) Kokwaro	Mumbu (Zi)	N	C, W	T	B	NT	Diarrhoea [15]
	*Mangifera indica* L.	Mwembe (Sw)	I	C	T	L	DD	Diarrhoea [15]
	*Ozoroa insignis* Delile	Mkala (Zi)	N	W	S	L	LC	Diarrhoea [15]
	*Searsia natalensis* (Bernh. ex C.Krauss) F.A.Barkley	Chegonde (Sa), Mkumba (Sw)	I	C	S	L	LC	Diarrhoea [15]

Annonaceae	*Uvaria acuminata* Oliv.	Mngwene (Zi/Sa)	N	C, W	S	R	LC	Convulsions [30]
	*Uvaria leptocladon* Oliv. subsp *leptocladon*	Mshofu (Sa/Zi)	N	C, W	S	R	NT	Epilepsy [30]
	*Uvaria lucida* Bojer ex Benth.	Mbwene (Sa/Zi)	N	C, W	S	F	LC	Measles [30]
	*Xylopia arenaria* Engl.	Mbaseri (Di)	N	W	S	R	VU	Convulsion [27]

Apocynaceae	*Rauvolfia vomitoria* Wennberg	Kinyabusinde (Hay)	N	C, W	S	B	LC	Malaria, splenomegaly and abdominal colics [38]
	*Strophanthus eminii* Asch. ex Pax	Masuchemengi (Zi/Sa)	N	W	T	B	—	Diarrhoea [34]

Araceae	*Stylochaeton natalense* subsp. *natalense*	Kigutwi (Zi)	N	W	H	R	—	Constipation, hernia and bloody cough [27]

Araliaceae	*Polyscias stuhlmannii* Harms	Konko (Zi)	N	W	S	L	EN	Diarrhoea [15, 34]

Arecaceae	*Phoenix reclinata* Jacq.	Ukindu (Sw)	N	W	T	L	LC	Diarrhoea [34]

Asparagaceae	*Asparagus africanus* Lam.	Lipalakanga (He)	N	W	S	R	—	Malaria, fever and convulsions [46]
	*Asparagus flagellaris* (Kunth) Baker	Kiandama (Sa)	N	W	S	L	—	Diarrhoea [15]
	*Asparagus racemosus* Willd.	Sangalechanda (Bar)	N	W	Cl	B	—	Calcium deficient [48]
	*Dracaena steudneri* Engl.	Mgologolo (Hay)	N	W	T	L	LC	Hernia, chest pain, asthma and splenomegaly [36, 37]

Asphodelaceae	*Aloe sp.*	Mlovera (Sw)	I	C	H	L	—	Intestinal worms [17]

Asteraceae	*Aspilia mossambicensis* (Oliv.) Wild.	Kashenganzili (Hay), Suruwa (Sw)	N	W	H	R, L	—	Convulsions [22] and retarded growth [31]
	*Acmella caulirhiza* Delile	Mtango (Pa)	I	C	H	L	LC	Convulsions [23]
	*Bidens pilosa* L.	Ndasa (Ny)	I	C	H	L	—	Wound and relapsing fever [20, 44]
	*Gutenbergia polycephala* Oliv. & Hiern	Akatoma (Hay)	N	W	H	L	—	Prevent belching [37]
	*Emilia javanica* (Burm.f.) C.B.Rob.	Kamuguze (Za)	I	C	H	L	—	Vomiting and diarrhoea [23]
	*Jeffreycia hildebrandtii* (Vatke) H.Rob., S.C.Keeley & Skvarla	Muluka (Sa)	I	C	S	R	—	Convulsions [23]
	*Launaea cornuta* (Hochst. ex Oliv. & Hiern) C.Jeffrey	Kihawa (Lu), Mchunga (Sw)	N	C, W	H	L	—	Convulsions [23]
	*Microglossa pyrrhopappa* (Sch.Bip. ex A.Rich.) Agnew	Mshashu (Sa)	N	W	S	R	—	Convulsions [30]
	*Microglossa pyrifolia* (Lam.) Kuntze	Kichuaghembe (Pa)	N	W	S	Wh	—	Fever, heartburn and epilepsy [30]
	*Pluchea dioscoridis* (L.) DC.	Mnywenywe (Zi)	N	C, W	S	L	LC	Convulsions [30]
	*Solanecio angulatus* (Vahl) C.Jeffrey	Leza (Sa)	I	C	S	L	—	Malarial convulsions (cerebral malaria) [30]
	*Solanecio mannii* (Hook.f.) C.Jeffrey	Akagango‐akake (Hay)	I	C	H	Ap	LC	Febrile convulsions [38]

Bignoniaceae	*Kigelia africana* (Lam.) Benth.	Nyejeza (Zi)	N	C, W	T	L	LC	Diarrhoea [15]
	*Markhamia obtusifolia* (Baker) Sprague	Muyuyu (Mak)	N	W	T	R	LC	Convulsions [30]
	*Markhamia zanzibarica* (Bojer ex DC.) K.Schum.	Mtalawanda (Za)	N	W	S	R	LC	Constipation [27]
	*Stereospermum kunthianum* Cham.	Mgunku (Zi)	N	W	S	B	LC	Diarrhoea [15]

Boraginaceae	*Ehretia amoena* Klotzsch	Mkilika (Sw)	N	C, W	S	L	LC	High fever [19]
	*Ehretia cymosa* var. *silvatica* (Gürke) Brenan	Leza (Sa)	N	W	S	L	LC	Diarrhoea [15]

Burseraceae	*Commiphora pteleifolia* Engl.	Mtwintwi (Zi)	N	W	S	L, B	NT	Diarrhoea [15]

Celastraceae	*Elaeodendron schlechterianum* (Loes.) Loes.	Mnenekanda (Sw/Zi)	N	W	S	L, B	LC	Diarrhoea [15]
	*Salacia Stuhlmanniana* Loes.	Sutang’andu (Zi)	N	W	S	L	—	Diarrhoea [15]

Cleomaceae	*Cleome gynandra* L.	Mgagani (Sw)	N	C, W	H	L	—	Retarded growth [31]

Combretum	*Combretum collinum* Fresen	Mlama ng’ombe (Zi)	N	W	T	B	LC	Diarrhoea [15]
	*Combretum pisoniiflorum* (Klotzsch) Engl.	Mlama mweusi (Sw/Zi, Pa)	N	W	T	R, L, B	LC	Diarrhoea [15, 34]
	*Terminalia sambesiaca* Engl. & Diels	ns	N	C, W	T	L	LC	Cold and fever [32, 33]

Connaraceae	*Rourea coccinea* subsp. *boiviniana* (Baill.) Jongkind	Mhamvi (Kw)	N	W	S	R	LC	Convulsions [23]

Crassulaceae	*Kalanchoe crenata* (Andrews) Haw.	Lipolopolo (He)	I	C	H	L	—	Spleen pains [46]
	*Kalanchoe pinnata* (Lam.) Pers.	Chikugwa (Hay)	I	C	H	L	—	Cold, flu and cough [38]

Cucurbitaceae	*Zehneria scabra* subsp. *argyrea* (Zimm.) C.Jeffrey	Akabindizi (Hay)	N	W	Cl	L	—	Skin diseases [38]
	*Citrullus lanatus* (Thunb.) Matsum. & Nakai	Ns	I	C	H	Tw	—	Early walk [23]

Dioscoreaceae	*Dioscorea praehensilis* Benth.	Engikwa (Ma)	N	C, W	Cl	R	LC	Rheumatism [5]

Ebenaceae	*Diospyros fischeri* Gürke	Mgotu (Zi)	N	W	S	R	LC	Diarrhoea [15]
	*Diospyros loureiroana* subsp. *loureiroana*	Kinyalinyali, Mguto (Zi)	N	W	H	L	LC	Measles and Malaria [23]

Euphorbiaceae	*Acalypha petiolaris* Hochst.	Umugonampiri (Tu)	N	W	H	R	—	Painful micturition [43]
	*Erythrococca kirkii* (Müll.Arg.) Prain	Mnyembeule (Zi), Mhumba (Kw)	N	W	S	R	—	Fever and convulsions [24, 28]

Fabaceae	*Albizia anthelmintica* (A.Rich.) Brongn.	Mfuleta (Sw)	N	W	T	B	LC	Diarrhoea [15] and Taenia [28]
	*Abrus precatorius* L.	Kaligaligo (Hay), Kitinutimu (Sw), Lufambo, (Pa, Za, Zi)	N	C, W	Cl	L	—	Coughs [21, 38]
	*Brachystegia spiciformis* Benth.	Mzinghawa nyika (Lu)	N	W	T	R	LC	Fever [18]
	*Dalbergia melanoxylon* Guill. & Perr.	Mpingo (Sw)	N	W	T	L	NT	High fever [19, 32, 33]
	*Cassia abbreviata* Oliv.	Mkundekunde (Sw)	N	W	T	R	LC	Diarrhoea [15, 19]
	*Erythrina abyssinica* Lam.	Mnungumagoma (Zi)	N	W,C	T	R	LC	Diarrhoea [15]
	*Indigofera swaziensis* Bolus	Mharashambuzi (Zi)	N	W	S	R	—	Diarrhoea [15]
	*Indigofera hirsuta* L.	Mpangipangi (He)	N	W	T	L	—	Infant complications [19]
	*Vachellia gerrardi* (Benth.) P.J.H.Hurter	Mkongowe (Zi)	N	W	T	R	—	Diarrhoea [15]
	*Millettia lasiantha* Dunn	Mhafie (Sa)	N	W	Cl	B	—	Diarrhoea [15]
	*Sesbania microphylla* Harm.	Msenga (Hay)	N	W	H	L	—	Malaria and febrile convulsions [36]
	*Senegalia polyacantha* (Willd.) Seigler & Ebinger	Mgunga (Zi)	N	W	T	L, R	LC	Sores [28]
	*Senna singueana* (Delile) Lock	Mvumba (Sw)	N	W	T	R	LC	Convulsions [30]
	*Tamarindus indica* L.	Mkwaju (Sw)	I	C	T	L	LC	Diarrhoea [15]
	*Vachellia nilotica* (L.) P.J.H.Hurter & Mabb.	Mtusi (Zi)	N	W	S	B	LC	Diarrhoea [15]
	*Zenkerella grotei* (Harms) J.Léonard	Mfundofundo (Sa), Kifundo (Sw)	N	W	T	L	—	Diarrhoea [15]

Hypericaceae	*Psorospermum febrifugum* Spach	Makandiza (Zi)	N	W	S	L	LC	Diarrhoea [15]

Lamiaceae	*Coleus barbatus* (Andrews) Benth. ex G.Don	Agacuncu (Han), Mvugh’va (Sa)	N	C, W	S	L	—	Stomach ache, constipation, measles [43] and measles [28]
	*Clerodendrum capitatum* (Willd.) Schumach.	Kapolo (Ny)	N	W	S	R	LC	Constipation [44]
	*Hoslundia opposita* Vahl.	Omunyenyete (Kur), Mvuma/Mtambaajongoo (Sa)	N	C, W	S	R, L	—	Fever and Convulsions [28, 35]
	*Kuloa usambarensis* (Engl.) Trofimov & Rohwer	Mseri (Sw)	N	W	T	B	—	Stomach ache [19] and infant complications [32, 33]
	*Leonotis ocymifolia* var. *raineriana* (Vis.) Iwarsson	Kitalelante (Hay)	N	W	H	L	—	Stomach aches and convulsions [22]
	*Ocimum suave* Willd.	Kivumbasi (Sa), Mzumbasha (Zi)	N	C	S	L	—	Diarrhoea [15] and colic [45]
	*Melissa officinalis* L.	Nanaa (Sw)	I	C	H	L	LC	Cold [39]
	*Mesosphaerum suaveolens* (L.) Kuntze	Kifumbasi (Sa), Kamba (Cha), Mfumbazi (Zi), Mhingajini (Za)	I	C	H	L	—	Convulsions [24, 28]
	*Mesosphaerum pectinatum* (L.) Kuntze	Hozandongole (Bo, Zi)	I	C	H	Wh	—	Intestinal worms [24]

Loganiaceae	*Strychnos henningsii* Gilg	Olduyesi (Ma)	N	W	S	B	LC	Colic [5]
	*Strychnos innocua* Delile	Tungotungo (Zi)	N	W	S	R	LC	Diarrhoea [15]
	*Strychnos spinosa* Lam.	Orurema (Hay)	N	W	S	L	LC	Malaria [40]

Malvaceae	*Dombeya rotundifolia* (Hochst.) Planch.	Mlwati, Mchimbu (Zi)	N	W	S	R, L, B, Co	LC	Diarrhoea [15, 39]
	*Grewia forbesii* Harv. ex Mast.	Mkole (Sw), Mkongodeka (Zi)	N	W	T	R	—	Convulsions [29]
	*Grewia microcarpa* K.Schum.	Mkongodeka	N	W	S	L	—	Diarrhoea [15]
	*Hibiscus micranthus* L.f.	Msase (Sa), Muambe (Sw), Muharasha‐mbuzi (Zi)	N	W	S	R	—	Convulsive fever [24, 28]
	*Waltheria indica* L.	Ns	I	C, W	T	L	LC	Convulsions [29]

Menispermaceae	*Cissampelos pareira* L.	Chegonde (Sa)	N	C, W	H	R	—	Pimples [28]

Monimiaceae	*Xymalos monospora* (Harv.) Baill.	Mvungawiza (Sa), Kamagaliko (Hay)	N	W	S	R	LC	Diarrhoea [15] and cough [38]

Moraceae	*Ficus sycomorus* L.	Mkuyu (Sw)	N	W	T	Sb	LC	Sore throat [21]
	*Milicia excelsa* (Welw.) C.C.Berg	Mvule (Sw)	N	W	T	B	NT	High fever [19]

Moringaceae	*Moringa oleifera* Lam.	Mlonge (Sw)	I	C, W	T	L	LC	Anaemia [47]

Myricaceae	*Myrica salicifolia* Hochst. ex A.Rich.	Mshengeshe (Sa)	I	C	T	L	LC	Diarrhoea [15] and tonic [28]

Myrtaceae	*Psidium guajava* L.	Mpera (Sw)	I	C	T	L, Tw	LC	Malaria and fever [22], diarrhoea [15, 21]

Ochnaceae	*Brackenridgea zanguebarica* Oliv.	Mkatakwa (Zi)	N	W	S	B	—	Diarrhoea [15]
	*Ochna atropurpurea* DC.	Mtingwa (Za)	N	W	S	R	—	Gangrenous rectitis [21]
	*Ochna holstii* Engl.	Mkumbi (Sw/Zi)	N	W	T	R, L, B	LC	Diarrhoea [15]

Olacaceae	*Ximenia americana* L.	Mtundwi (Zi), Mtundwa (Su)	N	W,C	S	R, L	LC	Diarrhoea [15], trachoma [28] and convulsions [21]

Oleaceae	*Jasminum fluminense* Vell.	Muhafu (Za), Mwangalaya (Zi)	N	W	S	L	—	Intestinal worms [21]

Peraceae	*Clutia abyssinica* Jaub. & Spach	Mhende (Sa)	N	W	S	L, R	LC	Sores [28]

Phyllanthaceae	*Antidesma venosum* E.Mey. ex Tul.	Inyamaza (Vi), Mjembajemba (Za), Mtanda na mbingu (Nd)	N	C, W	T	R	LC	Purulent coughs [25]
	*Flueggea virosa* (Roxb. ex Willd.) Royle	Mkwambe (Nd, Sw, Za)	N	W	T	L, R	LC	Abdominal pains [19, 24]

Poaceae	*Saccharum officinarum* L.	Maji ya miwa (Sw)	I	C, W	S	St	—	Asthma [39]

Polygalaceae	*Carpolobia goetzei* Gürke	Mzukizuki (Za)	N	C, W	S	R	LC	Constipation [26]
	*Securidaca longepedunculata* Fresen.	Masuke mengi (Zi), MSigi (Za), Nengonengo (Su)	N	W	T	Sb	LC	Convulsions [26]

Polygonaceae	*Rumex usambarensis* (Engl.) Dammer	Nyanywa (Sa)	N	W	S	L	—	Constipation [29]

Polypodiaceae	*Dryopteris inaequalis* (Schltdl.) Kuntze	Kilaho (Cha)	N	W	H	Rh	—	Roundworms [27]

Rubiaceae	*Catunaregam nilotica* (Stapf) Tirveng.	Mdasha (Zi), Mpigi (Nd), Mtutuma (Za), Mwachanguku (Su)	N	W	T	R	—	Convulsions [26], hernia [19]
	*Multidentia fanshawei* (Tennant) Bridson	Mdegedege (Lu)	N	W	T	R	—	Fever [18]
	*Chassalia umbraticola* Vatke	Uvuguro (Za), Mliya (Nd)	N	W	S	R	LC	Convulsions [26]
	*Chassalia violacea* K.Schum.	Mdunula (He)	N	W	S	R	EN	Infant complications [19]
	*Hymenodictyon parvifolium* Oliv.	Mazi ge Nkeremeke (Hay)	N	W	T	Ap	LC	Malaria and chest problems involving breath difficulties [38]
	*Keetia zanzibarica* (Klotzsch) Bridson	Horohozo, Tindigala (Za)	N	W	S	R	—	Convulsions [26]
	*Pyrostria bibracteata* (Baker) Cavaco	Mdavidavi (Zi)	N	W	T	R	LC	Convulsions [26]
	*Rytigynia uhligii* (K. Schum. & K. Krause) Verdc.	Mtulavuha (Zi)	N	W	S	R	LC	Diarrhoea [15]
	*Vangueria infausta* Burch.	Mviru (Zi), Msada (He, Lu)	N	C, W	S	R	LC	Swelling of genital organs and legs [29], infant complications [19]

Rutaceae	*Clausena anisata* (Willd.) Hook.f. ex Benth.	Mvuje pori (Kw/Sw)	N	W	S	L	LC	Colic [41]
	*Harrisonia abyssinica* Oliv.	Mkusu (Sw), Mzuma (Sa)	N	W	S	L	LC	Diarrhoea [15]
	*Zanthoxylum capense* (Thunb.) Harv.	Mlungulungu (Sw)	N	W	S	B	LC	Infant complications [19]
	*Zanthoxylum deremense* (Engl.)	Mlungulungu (Lu)	N	W	S	Fr	VU	Fever [18]
	*Zanthoxylum chalybeum* Engl.	Mnukapala (Sa)	N	W	S	L	LC	Diarrhoea [15] and appetite [19, 29]

Sapindaceae	*Allophylus rubifolius* (Hochst. ex A.Rich.) Engl.	Msembele (Zi), Msempelele (Za), Mhecha (Ng)	N	W	S	R	LC	Diarrhoea [29], anaemia and constipation [26]
	*Blighia unijugata* Baker	Mzindanguwe (Sa)	N	C, W	T	L	LC	Diarrhoea [15]
	*Deinbollia borbonica* Scheff.	Mhaikanyoya, Mkomanguku (Zi)	N	W	S	R	LC	Diarrhoea [15], stomach ache and vomiting [29], convulsions [26]
	*Zanha africana* (Radlk.) Exell	Mdaula (Sw/Zi)	N	C, W	T	R	—	Diarrhoea [15] and flu [39]

Sapotaceae	*Inhambanella henriquesii* (Engl. & Warb.) Dubard	Kongukubwa (Zi)	N	W	T	L	—	Diarrhoea [15]

Solanaceae	*Capsicum frutescens* L.	Pilipili kichaa (Sw)	I	C	H	R	LC	Convulsions [25]
	*Solanum anguivi* Lam.	Avitamo (Ira)	N	C, W	S	L	LC	Sand fleas [42]
	*Solanum incanum* L.	Ndulele (Sw), Mtula (Zi/Sa)	N	C, W	H	R	LC	Diarrhoea [15, 34]
	*Solanum nigrum* L.	Shwiga (Hay)	I	C, W	H	L, Fr, R	—	Bed wetting [36, 38]
	*Withania somnifera* (L.) Dunal	Lifubefube (Ji)	N	C, W	S		DD	Convulsions [35]

Thymelaeaceae	*Synaptolepis kirkii* Oliv.	Mvungaliza (Zi)	N	W	S	L	LC	Diarrhoea [15]

Verbenaceae	*Lantana camara* L.	Omuuki (Hay)	I	C, W	S	F	—	Cough [37]
	*Lantana trifolia* L.	Kashekelauku (Hay),	I	C, W	S	L	—	Febrile convulsions [25, 38]

Vitaceae	*Cissus aralioides* (Welw. ex Baker) Planch.	Hoza (Sw/Zi)	N	W	Cl	L	—	Diarrhoea [15]
	*Cyphostemma njegerre* (Gilg) Desc.	Mwengele (Zi)	N	W	H	R	—	Diarrhoea [15]

*Note:* Local names∗: Sw = Swhili, Zi = Zigua, Sa = Sambaa, Pa = Pare, Hay = Haya, Ji = Jita, Lu = Luguru, Tu = Tusti, Ny = Nyamwezi, Kw = Kwere, Ma = Maasai, Mak = Makonde, Cha = Chaga, Di = Digo, Za = Zaramo, Nd = Ndengereko, Bo = Bondei, Su = Sukuma, Ng = Ngindo, Vi = Vidunda, He = Hehe, Bar = Barbeig, Ira = Iraq and ns = not specified. OG = originality: N = native, I = introduced. So = sources of plants: W = wild and C = cultivated. LF = life form: T = tree, H = herb, S = shrub, Cl = climber, PU = part used: R = roots, L = leaves, B = barks, Co = cords, Tw = twigs, Fr = fruits, Ap = aerial parts, F = flowers, Sb = stem bark, Rb = root bark, St = stem, Wh = whole plant, Rh = rhizomes, CS = conservation status: EN = endangered, VU = vulnerable, NT = near threatened, DD = data deficient and LC = least concern.

### 3.3. Life Forms and Parts Used

**FIGURE 2:**
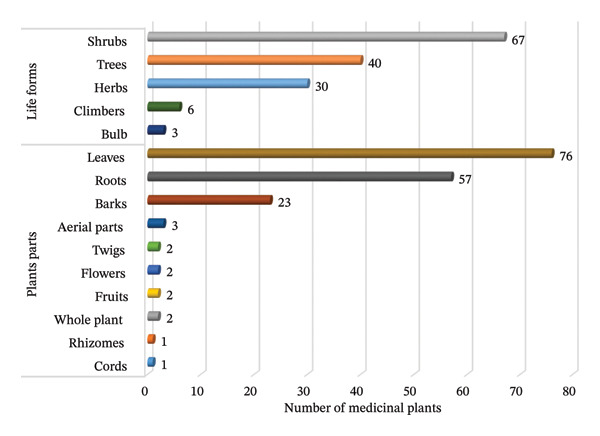
Life forms and plant parts used for the preparation of paediatric herbal remedies.

Shrubs (46.0%) accounted for the highest proportion of MPs, followed by trees (27.4%), herbs (20.5%), climbers (4.1%) and bulbs (2.1%) (Figure 2). The high utilisation of shrubs reflects their abundance in the regions and locals’ knowledge of their use to treat and manage various human ailments. These findings contradict those of Tugume and Nyakoojo [13] and Maroyi [50], who reported that herbs were the dominant life form for PAs in Uganda and Zimbabwe, respectively. Shrubs and trees are preferred life forms for treatment because they are not affected by seasonality and are readily available and accessible year‐round, thereby guaranteeing sustainable management of PAs [52, 53].

The plant parts used to prepare PA’s herbal remedies include leaves, roots, bark, fruits, twigs and aerial parts. In some cases, whole plants are also utilised. Leaves (52.1%) are the most commonly used part, followed by roots (39.0%), and bark (15.8%). The use of other parts amounts to 2% or less (Figure 2). The extensive use of leaves may be attributed to their effectiveness, including greater accumulation of bioactive ingredients with therapeutic potential, ease of harvesting and rapid regenerative capacity [54, 55]. Leaves serve as the primary photosynthetic organs in plants and are considered a natural pharmacy for synthesising many active constituents, including secondary metabolites with pharmacological activity against various human ailments [56, 57]. Similarly, the widespread utilisation of leaves in the preparation of PAs has been reported in Nigeria [58], Zimbabwe [50], Uganda [13] and Morocco [59]. Although roots are the second most utilised part after leaves for making herbal remedies for PAs, they are believed to contain extra potent pharmacological ingredients. However, their exploitation should be done cautiously, as it can destroy and jeopardise MPs’ survival [60, 61]. Therefore, using leaves is encouraged whenever possible, as they are readily available, easy to harvest and simple to formulate into herbal remedies.

Although the majority (80.1%) of the plant parts were used singly, some instances involved multiple parts of the same plant. For example, the roots and leaves of *Clutia abyssinica* Jaub. & Spach (Peraceae) and *Flueggea virosa* (Roxb. ex Willd.) Royle (Phyllanthaceae) were used to manage sores and abdominal pains, respectively. Leaves and bark of *Commiphora pteleifolia* Engl. (Burseraceae) and *Elaeodendron schlechterianum* (Loes.) Loes. (Celastraceae) were used to treat diarrhoea. The roots, leaves and bark of *Ochna holstii* Engl. (Ochnaceae) and *Combretum pisoniiflorum* (Klotzsch) Engl. (Combretaceae) were used to manage diarrhoea. Furthermore, the whole plant of *Microglossa pyrifolia* (Lam.) Kuntze (Asteraceae) and *Mesosphaerum pectinatum* (L.) Kuntze (Lamiaceae) was used to manage fever, epilepsy, heartburn and intestinal worms.

### 3.4. Originality and Sources of MPs

**FIGURE 3:**
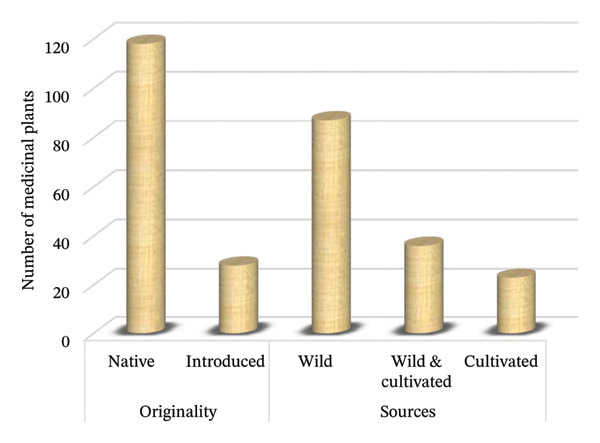
Origins and sources of medicinal plants.

Among the recorded MPs in this review, 80.8% were native species, while 19.2% were introduced species (Figure 3). Moreover, the study shows that 59.6% of the MPs are gathered from wild ecosystems (general lands and forest reserves), 15.8% from cultivated ecosystems (farms and home gardens) and 24.7% from both wild and cultivated ecosystems (Figure 3). The results suggest that wild ecosystems, especially forest reserves, are essential for the livelihood of locals in the country. However, the cultivation system appears weak, with increased pressure to exploit wild resources. This finding aligns with ethnobotanical studies from the Suro Barguda district in Ethiopia [56] and the Rukungiri district in Uganda [13], which show that most MPs are well‐adapted and available in wild ecosystems but are highly affected by the overharvesting for diverse uses. Therefore, cultivating valuable MHs in farms and home gardens is highly recommended for sustainability.

### 3.5. Disease Categories

This review revealed that the 146 recorded MPs are used to treat and manage 51 PAs. The ailments were categorised into 15 disease categories (Table 3), with gastrointestinal disorders, neurological disorders, malaria/fever, respiratory disorders, dermatological disorders and pains being treated by the largest number of MPs. Among the PAs treated by the most MPs were diarrhoea (51 species), convulsions (32 species), malaria and fever (25 species) and coughs and flu (10 species). Nine species were recorded for treating and managing at least three ailments: *Rauvolfia vomitoria* Wennberg (Apocynaceae), *Stylochaeton natalense* subsp. *natalense* (Araceae), *Dracaena steudneri* Engl. (Asparagaceae), *Asparagus africanus* Lam. (Asparagaceae), *Microglossa pyrifolia* (Lam.) Kuntze (Asteraceae), *Coleus barbatus* (Andrews) Benth. ex G.Don (Lamiaceae), *Ximenia americana* L. (Olacaceae), *Deinbollia borbonica* Scheff. (Sapindaceae), and *Allophylus rubifolius* (Hochst. ex A.Rich.) Engl. (Sapindaceae). In line with this review’s findings, a study conducted in Indonesia revealed that herbal remedies effectively manage diarrhoea in children [62]. MPs’ overall role and value in children’s welfare are emphasised through applying several MPs in managing PAs (Table 2). Therefore, the locals in Tanzania have vast knowledge and skills in exploiting MPs for treating and managing PAs.

**TABLE 3:** List of disease categories treated by medicinal plants.

Disease category	Number of MPs (*N* = 146)	% of the total MPs
Gastrointestinal disorders	63	43.2
Neurological disorders	34	23.3
Malaria and fever	25	17.1
Respiratory disorders	16	11.0
Dermatological disorders	6	4.1
Pains	5	3.4
Splenomegaly	3	2.1
Measles	3	2.1
Hernia	3	2.1
Retard growth	2	1.4
Anaemia	2	1.4
Bed wetting	1	0.7
Rheumatism	1	0.7
Chicken pox	1	0.7
Appetite	1	0.7

Moreover, it should be noted that some of the PAs were treated and managed using a single MP, while in other few cases, a mixture of MP parts from different species was employed; however, monotherapy preparations predominated compared to herbal concoctions. For instance, constipation was treated by the roots of *Clerodendrum capitatum* (Willd.) Schumach. (Lamiaceae) and leaves *Rumex usambarensis* (Engl.) Dammer (Polygonaceae); measles and malaria were treated using leaves of *Diospyros loureiroana* subsp. *loureiroana* (Ebenaceae); convulsions using leaves of *Pluchea dioscoridis* (L.) DC. (Asteraceae) and roots *Chassalia umbraticola* Vatke (Rubiaceae); diarrhoea using leaves of *Psorospermum febrifugum* Spach (Hypericaceae) and roots of *Psorospermum febrifugum* Spach (Loganiaceae); and intestinal worms using the whole plant of *Mesosphaerum pectinatum* (L.) Kuntze (Lamiaceae).

### 3.6. Literature Supporting Traditional Use in Other Countries

Among the recorded MPs in this review, some have also been documented in other African countries, where they are used to treat and manage various PAs. For instance, in Uganda, the leaves of *Ocimum suave* Willd. (Lamiaceae) are reported to treat stomach aches; the leaves and bark of *Mangifera indica* L. (Anacardiaceae) are used for coughs, oral wounds, tonsillitis and fevers; the leaves of *Lantana trifolia* L. (Verbenaceae) are utilised to treat fresh wounds; and the leaves and bark of *Psidium guajava* L. (Myrtaceae) are used to prepare herbal remedies for coughs, diarrhoea, fever, malaria and anaemia [13, 63]. In South Africa, the roots and leaves of *Withania somnifera* (L.) Dunal (Solanaceae) treat and manage constipation, sores or pulse, restlessness and a sunken fontanelle; the leaves, stem and rhizomes of *Aloe arborescens* Mill. (Xanthorrhoeaceae) and *Aloe maculata* All. (Xanthorrhoeaceae) treat and manage skin itching, irritation, umbilical cord disorders, urinary tract infections, burns and diarrhoea [64]. In Nigeria, the leaves of *M. indica* treat fever, malaria and jaundice; the whole plant of *Allium sativum* L. (Amaryllidaceae) treats coughs; the entire plant of *Cleome gynandra* L. (Cleomaceae) treats and manages pneumonia; the fruits of *Tamarindus indica* L. (Fabaceae) help with bed wetting; the entire plant of *Waltheria indica* L. (Malvaceae) is used for teething; the leaves of *P. guajava* are used to treat headaches and malaria; the roots of *Moringa oleifera* Lam. (Moringaceae) treat malaria and bilharzia; the roots of *Ximenia americana* L. (Olacaceae) are used to treat and manage kwashiorkor; and the leaves of *Clerodendrum capitatum* (Willd.) Schumach. (Lamiaceae) are used against pneumonia, ear infections and sickle cell anaemia [58].

In addition, in Zimbabwe, the roots of *Asparagus africanus* Lam. (Asparagaceae) are used to treat constipation; the leaves of *Bidens pilosa* L. (Asteraceae) are employed for wound treatments; the roots of *Combretum pisoniiflorum* (Klotzsch) Engl. (Combretaceae) are utilised to address depressed fontanelle and help fatten infants; the roots of *Erythrina abyssinica* Lam. (Fabaceae) are used to manage wasting; the roots of *Ficus sycomorus* L. (Moraceae) are used for depressed fontanelle; the roots of *Hoslundia opposita* Vahl. (Lamiaceae) are used to treat and manage diarrhoea, gastroenteritis and abdominal pain; the leaves, fruits and seeds of *M. oleifera* combat malnutrition; the roots of *Ozoroa insignis* Delile are utilised against inflammation of the umbilical cord; the roots of *Senna singueana* (Delile) Lock (Fabaceae) are used to treat and manage umbilical cord removal, constipation and abdominal pain; the leaves and roots of *Solanum incanum* L. (Solanaceae) are used against depressed fontanelle and wasting; and the roots of *X. americana* treat and manage diarrhoea and abdominal pain [50]. Such similarities in MP uses, beliefs and values highlight the importance of documenting aboriginal and local knowledge about MPs across different cultures and geographical settings. The insights from these ethnomedicinal applications and cross‐cultural appraisals offer promising avenues for advanced ethnopharmacological investigation.

### 3.7. Pharmacological Evidence Against PAs

Of the recorded MPs in this review, 92 (63.0%) have been investigated for their phytochemical content and exhibit various pharmacological activities (Table 4). The majority of MPs in this review (Table 3) exhibit multiple pharmacological activities, including antimicrobial, antibacterial, antioxidant, antidiabetic, antiviral, antifungal, anticancer, anti‐inflammatory, antiplasmodial, hypoglycaemic, immunostimulatory, immunomodulatory and cardioprotective effects (Table 4). The primary phytochemical constituents are flavonoids (79.3%), followed by phenols (63.0%); saponins and alkaloids (58.7% each); tannins (50.0%); terpenoids, glucosides and steroids (33.7% each); quinones (17.4%); and triterpenes (14.1%) (Table 4). This review supports the findings of Maroyi [179], which indicate that flavonoids are the predominant secondary metabolites in the MPs of Zimbabwe. Similarly, the study conducted by Wink [180] suggests that alkaloids, phenolics and terpenoids represent significant secondary metabolites in herbal remedies. Despite the identification of many secondary metabolites, MPs have received little ethnopharmacological attention. Over 40% of these species are commercially substantial in both informal and formal markets across Africa [31, 179, 180]. This underscores the economic importance of our research, as many species are widely used for traditional remedies in Zimbabwe [181], South Africa [182], Zambia [183], Mozambique [184] and Namibia [185]. Various ethnobotanical studies conducted elsewhere show that some MPs are commercially exploited for various ailments. Notable MPs with pharmaceutical potential that are commercially exploited include Abrus precatorius L. (Fabaceae), E. abyssinica and *Dalbergia melanoxylon* Guill & Perr. (Fabaceae)*, Cassia abbreviata* Oliv. (Fabaceae)*, Kigelia africana* (Lam) Benth. (Bignoniaceae)*, B. pilosa, Milicia excelsa* (Welw.) C.C.Berg (Moraceae) and *T. indica* [31, 183, 185, 186].

**TABLE 4:** Literature on pharmacological activities and phytochemical constituents of some recorded medicinal plants.

Species name	Pharmacological activities	Phytochemical constituents	References
*Abrus precatorius* L.	Antihyperlipidaemia, abortifacient, antidiabetic, antiviral, antifertility, anti‐inflammatory, antimicrobial, antioxidant, antiepileptic, antimalarial, antitumour, immunostimulatory, antiparasitic, neuro‐ and nephron‐protective	Saponins, phenolics, alkaloids, flavonoids, glycosides, steroids and terpenoids	[65, 66]
*Achyranthes aspera* L.	Antiperiodic, purgative, laxative, antiviral, antiasthmatic, hepatoprotective, antiallergic, antitumour, antiplasmodial, antihypertensive, anticoagulant and antidiuretic	Alkaloids, tannins, saponins, flavonoids and glycosides	[67, 68]
*Acmella caulirhiza* Delile	Antitumour, analgesic, anti‐inflammatory, antibacterial and antioxidant	Phenols, sterols, alkaloids, flavonoids, tannins and coumarins	[69, 70]
*Aeschynomene elaphroxylon* (Guill. & Perr.) Taub.	Antioxidant, anti‐inflammatory and antimicrobial	Triterpenoid and saponins	[71]
*Albizia anthelmintica* (A.Rich.) Brongn.	Antioxidant and cytotoxic	Phenols	[72]
*Allium sativum* L.	Antiviral, anticancer, antibacterial, antifungal, antidiuretic, antiparasitic, antidiabetic, anti‐inflammatory, antioxidant, hypolipidemic, hepato‐, radio‐ and cardioprotective	Flavonoids	[73]
*Antidesma venosum* E.Mey. ex Tul.	Antibacterial, antimycobacterial, antifungal, antioxidant, anti‐inflammatory, antischistosomal and cytotoxic	Alkaloids, terpenoids, tannins, phytosterols, saponins, cardiac glycosides and flavonoids	[74]
*Aspilia mossambicensis* (Oliv.) Wild.	Antimicrobial, antioxidant, antihepatotoxic and hypoglycaemic	Flavonoids, alkaloids, saponin, steroids and anthraquinones	[75, 76]
*Bidens pilosa* L.	Antimalarial, antiallergy, antihypertensive, anticancerogenic, antihypertensive antifungal, antitumour, antidiabetic, antiulcerative, anti‐inflammatory, antimicrobial and antioxidant	Flavonoids, glycosides, phenolic acids, terpenoids and phytosterols	[77]
*Blighia unijugata* Baker	Antioxidant, antimicrobial, antidiabetic, anti‐inflammatory and anticancer	Coumarins, steroids, saponins and tannins	[78]
*Brackenridgea zanguebarica* Oliv.	Antiproliferative, antifungal, antibacterial, antiviral and antitumour	Flavonoids	[79]
*Capsicum frutescens* L.	Antioxidant, antimicrobial, analgesic, anticancer, antiseptic, immunomodulatory, anti‐inflammatory and counterirritant	Phenols, capsaicinoids and carotenoids	[80, 81]
*Cassia abbreviata* Oliv.	Antimicrobial, antibacterial, antidiabetic, antifungal, abortifacient, anti‐inflammatory, antimicrobial, antiviral, antioxidant and hepatoprotective	Tannins, phenols, alkaloids, flavonoids, anthraquinones and phenols	[82]
*Catunaregam nilotica* (Stapf) Tirveng.	Antioxidant	Saponins, flavonoids and phenols	[83]
*Chenopodium opulifolium* Schrad. ex W.D.J.Koch & Ziz	Antiviral, antimicrobial, antifungal, anti‐inflammatory, analgesic, antioxidant, anti‐nociceptive and immunomodulatory	Flavonoids, terpenes, tannins, alkaloids and saponins	[84]
*Cissampelos pareira* L.	Anti‐inflammatory, antioxidant, hepatoprotective, diuretic, antidiabetic and antiasthmatic	Alkaloids, tannins and triterpenes	[85]
*Cissus aralioides* (Welw. ex Baker) Planch.	Antibacterial and antimicrobial	Alkaloid, cardiac glycoside, flavonoid, terpenoids and saponins	[86, 87]
*Citrullus lanatus* (Thunb.) Matsum. & Nakai	Antibacterial, antifungal, antimicrobial, antiulcer, antioxidant, anti‐inflammatory, analgesic, laxative, antigiardial and gastro‐ and hepatoprotective	Alkaloids, steroids, saponins, glycosides, flavonoids, tannins and phenolic compounds	[88]
*Clausena anisata* (Willd.) Hook.f. ex Benth.	Antimicrobial, antioxidant, anti‐inflammatory, antitrypanosomal	Saponins, tannins, steroids, flavonoids, alkaloids, coumarins, phenols and glycosides	[89, 90]
*Cleome gynandra* L.	Antioxidant, antimicrobial, anti‐inflammatory anticancer, anti‐arthritic, antiparasitic, antimalarial, antifungal and cytotoxic	Phenols and flavonoids	[91, 92]
*Clerodendrum capitatum* (Willd.) Schumach.	Hypoglycaemic and hypolipidaemic	Terpenoids, glycosides, steroids and flavonoids	[93]
*Clutia abyssinica* Jaub. & Spach	Hepatoprotective, antioxidant, antimalarial, anti‐inflammatory, antipyretic and antitrypanosomal	Alkaloids, saponins, anthraquinones, phenolics, tannins, terpenoids and flavonoids	[94]
*Coleus barbatus* (Andrews) Benth. ex G.Don	Antiasthmatic, anti‐inflammatory, immunomodulatory, antimicrobial and antineoplastic	Terpenoids, phenols and alkaloids	[95, 96]
*Combretum collinum* Fresen	Antibacterial, anti‐inflammatory and antioxidant	Flavonoids and phenols	[97]
*Dalbergia melanoxylon* Guill. & Perr.	Antioxidant, antimicrobial, cytotoxic, antidiabetic, anticancer and anti‐inflammatory,	Flavonoids, tannins, alkaloids, quinones, phenols and glycosides	[98]
*Dioscorea praehensilis* Benth.	Antioxidant and antihyperglycemic	Polyphenols, tannins, steroids, quinones, flavonoids, anthocyanins and terpenoids	[99]
*Dombeya rotundifolia* (Hochst.) Planch.	Antibacterial, anti‐inflammatory, anthelmintic, antioxidant, antihypertensive and acetylcholinesterase inhibitory	Saponins, tannins, cardiac glycosides, flavonoids, phenolics, steroids and terpenoids	[100]
*Dracaena steudneri* Engl.	Anti‐inflammatory, antiallergenic, antiviral, antioxidant, anticarcinogenic, cardiotonic, antidiabetic, antifungal, antiplasmodial, oxytocic, antiprotozoal and antimicrobial	Alkaloids, flavonoids, terpenoids, saponins, tannins, glycosides and phenols	[101]
*Ehretia amoena* Klotzsch	Antiprotozoal, antitrypanosomal, cytotoxic and anthelmintic	Saponins, steroids, tannins, anthraquinones and terpenoids	[102]
*Elaeodendron schlechterianum* (Loes.) Loes.	Antibacterial, antiparasitic, anti‐inflammatory and anti‐HIV	Phenols and terpenoids	[103]
*Erythrina abyssinica* Lam.	Anti‐inflammatory, antibacterial, antioxidant, antifungal, antiplasmodial, antiproliferative, antimycobacterial, antidiarrheal, anti‐HIV 1, antidiabetic and antiobesity	Flavonoids, alkaloids and terpenoids	[104]
*Ficus sycomorus* L.	Antifungal, antimicrobial and cytotoxic	Tannins, saponins, alkaloids, glycosides and flavonoids	[105]
*Flueggea virosa* (Roxb. ex Willd.) Royle	Cytotoxic, antiparasitic, antimicrobial, antioxidant, antidiabetic, aphrodisiac, laxative, analgesic, anti‐inflammatory, antimalarial, anti‐HIV, anti‐hepatitis C, antiarrhythmic, antidiarrheal, sedative and trypanocidal, antiparasitic and anticancer.	Phenols, terpenoids, alkaloids, tannins, saponins, cardiac glycosides and steroidal	[106, 107]
*Harrisonia abyssinica* Oliv.	Antimicrobial, antiviral, antifungal and molluscicidal	Saponins, tannins, flavonoids alkaloids and terpenoids	[108, 109]
*Hibiscus micranthus* L.f.	Antibacterial, antioxidant, cytotoxic, antidiabetic	Alkaloids, flavonoids, saponins, diterpenes, tannins, steroids, phenols and anthraquinones	[110, 111]
*Hoslundia opposita* Vahl.	Antibacterial, antiviral, antioxidant, antibiofilm and antifungal	Tannins, saponins, glycosides, phenols, alkaloids coumarins, triterpenoids, steroids and flavonoids	[112]
*Hymenodictyon parvifolium* Oliv.	Antimicrobial, antifungal, antibacterial	Flavonoids, terpenoids and glycosides	[113]
*Indigofera hirsuta* L.	Antioxidants and antimicrobial	Saponins, alkaloids, flavonoids, terpenoids, steroids and phenols	[114]
*Jasminum fluminense* Vell.	Mosquitocidal	Alkaloids, tannins, flavonoids, glycosides, phenols and saponins	[115]
*Kalanchoe pinnata* (Lam.) Pers.	Cytotoxic, anticancer, immunomodulatory, antiallergic, analgesic, antimicrobial, antihistamines, antitumour, antiviral and antibacterial	Flavonoids, terpenoids, steroids, alkaloids and glycosides	[116, 117]
*Kigelia Africana* (Lam.) Benth.	Antioxidant, anti‐inflammatory, antidiarrhoeal, antiulcer, analgesic, antiprotozoal and antibacterial	Flavonoids, phenolics, naphthoquinones, coumarins, tannins, saponins, glycosides and terpenoids	[118, 119]
*Kuloa usambarensis* (Engl.) Trofimov & Rohwer	Antibacterial	Flavonoids, phenols, saponins and tannins	[120]
*Lannea schweinfurthii* (Engl.) Engl.	Anti‐apoptotic, antibacterial, antiviral, antigiardial, anti‐inflammatory, antioxidant, antiplasmodial, antitrypanosomal, hepatoprotective and cytotoxic	Alkaloids, flavonoids, glycosides, phenols, saponins, steroids, tannins and terpenoids.	[121]
*Lantana camara* L.	Anticancer, antifilarial, nematocidal, antibacterial, antimutagenic, antileishmanial, antifungal, anti‐inflammatory and antioxidant	Terpenoids, flavonoids and glycosides	[122, 123]
*Lantana trifolia* L.	Antioxidants	Phenols and flavonoids	[124]
*Launaea cornuta* (Hochst. ex Oliv. & Hiern) C.Jeffrey	Antioxidant, antifungal, anti‐inflammatory	Saponin, phenols, flavonoids, alkaloids, terpenoids, anthraquinones and tannins.	[125]
*Mangifera indica* L.	Anticancer, antioxidant, antimicrobial, antidiarrheal, hepatoprotective	Phenolics and flavonoids	[126]
*Markhamia obtusifolia* (Baker) Sprague	Antimicrobial, cytotoxic and antifungal	Quinones and terpenoids	[127]
*Markhamia zanzibarica* (Bojer ex DC.) K.Schum.	Anti‐Alzheimer	Phytosterols and campesterols	[127]
*Melissa officinalis* L.	Cytotoxic, antioxidant, anti‐inflammatory, antifungal, antimicrobial, anticancer, antiviral, antiallergic, antitumour and antidiabetic	Flavonoids, terpenoids, phenolic acids, and tannins,	[128, 129]
*Mesosphaerum pectinatum* (L.) Kuntze	Anti‐inflammatory, antioxidant, anticancer, antimicrobial, antidepressant, hepatoregenerative and antinociceptive	Terpenes and flavonoids	[130]
*Mesosphaerum suaveolens* (L.) Kuntze	Antimicrobial, anti‐inflammatory, antioxidant, antibacterial and antifungal	Alkaloids, cardiac glycosides, flavonoids, saponins, triterpenes, diterpenes, tannins and phenols	[131, 132]
*Microglossa pyrifolia* (Lam.) Kuntze	Antibacterial, antifungal, antimalaria, antiasthmatic, immunomodulatory, anxiolytic and anticonvulsant	Flavonoids, alkaloids, glycosides, phenols, saponins, steroids and terpenoids	[133, 134]
*Milicia excelsa* (Welw.) C.C.Berg	Antihypoxic, antiplasmodial, cytotoxic and antioxidant	Alkaloids, phenols, flavonoids, saponins and tannins	[135, 136]
*Moringa oleifera* Lam.	Hepatoprotective, antihypertensive, cholesterol‐lowering, anti‐urolithiasis, antifertility, antidiabetic, antioxidant, nutraceutical and antimicrobial	Flavonoids, anthocyanins, steroids, alkaloids, saponins, terpenoids, anthraquinone and cardiac glycosides	[137]
*Myrica salicifolia* Hochst. ex A.Rich.	Molluscicidal	Alkaloids, flavonoids, steroids, terpenoids, tannins, saponins, quinones, phenols, glycosides and terpenoids	[138]
*Ochna holstii* Engl.	Antibacterial	Flavonoids	[139]
*Ocimum suave* Willd.	Antimicrobial	Tannins, flavonoids, saponins, steroids, alkaloids and phenolics	[140]
*Ozoroa insignis* Delile	Antimicrobial, anti‐inflammatory, anthelmintic and cytotoxic	Phenolics, tannins and flavonoids	
*Phoenix reclinata* Jacq.	Antiplasmodial, antipyretic and anti‐inflammatory	Glycosides, flavonoids, alkaloids, tannins, saponins, terpenoids, sterols and phenolics	[141]
*Pluchea dioscoridis* (L.) DC.	Antiaging, anticancer, anti‐inflammation, antiulcer, anti‐dyslipidaemia, antidiabetic, antinociceptive, antipyretic, antidiarrhoeal, antibacterial and antifungal	Terpenoids, phenolics, steroids and flavonoids	[142, 143]
*Psidium guajava* L.	Antidiabetic, antidiarrheal, hepatoprotective, anticancer, antioxidant, anti‐inflammatory, antimicrobial, antiallergy and antiplasmodial effects	Triterpenoids, alkaloids, steroids, tannins, glycosides, flavonoids and saponins.	[144]
*Psorospermum febrifugum* Spach	Antifungal, immunomodulatory, antipsoriatic, antitumour, antihepatotoxic, antioxidant, antimicrobial, antileukemia and anti‐inflammatory	Flavonoids, steroids, saponins, alkaloids, terpenoids, tannins and phenols	[145]
*Rauvolfia vomitoria* Wennberg	Antioxidant, antifungal, antibacterial and anticancer	Alkaloids, terpenoids, saponins, flavonoids, tannins and phenols	[146]
*Rumex usambarensis* (Engl.) Dammer	Appetite suppressing, antioxidant and antimicrobial	Flavonoids, benzopyranes, chromones and xanthones	[147]
*Saccharum officinarum* L.	Antimicrobial, anti‐inflammatory, analgesic, antioxidant, antihyperglycemic, diuretic and hepatoprotective Antithrombotic	Saponins, alkaloids, flavonoids, phenolics, phytosterols, terpenoids	[148, 149]
*Searsia natalensis* (Bernh. ex C.Krauss) F.A.Barkley	Antioxidant, cytotoxic, antibacterial, antifungal and antiulcer	Phenolics	[150, 151]
*Securidaca longepedunculata* Fresen.	Antibacterial	Alkaloids, flavonoids, saponins, steroids, tannins, cardiac glycosides and anthraquinones	[152]
*Senna singueana* (Delile) Lock	Antioxidant, antimalarial, anticancer, antibacterial, antidiabetic, antifungal, antinociceptive, hepatoprotective and trypanocidal	Steroids, flavonoids, tannins, phenols, anthraquinones and anthrones	[153]
*Solanecio mannii* (Hook.f.) C.Jeffrey	Cytotoxic and antibacterial	Alkaloids	[154]
*Solanum anguivi* Lam.	Antibacterial, antioxidant, antidiabetic and anti‐inflammatory	Flavonoids, phenolics, terpenoids, tannins, steroids, glycosides, saponins and alkaloids	[155, 156]
*Solanum incanum* L.	Antimicrobial and antioxidant	Alkaloids, saponins, flavonoids, glycosides, terpenoids and steroids.	[157]
*Solanum nigrum* L.	Immunomodulatory, anticancer, immunostimulant, antibacterial, antidiabetic, antiviral, anti‐inflammatory, antioxidant, antipyretic, antidiarrhoeal, neuro‐ and cardioprotective, anti‐hyperlipidaemic, hepatoprotective, antiallergic and antiasthmatic	Steroids, saponins, alkaloids and phenols	[158, 159]
*Stereospermum kunthianum* Cham.	Antibacterial, antioxidant, antiplasmodial, analgesic, anti‐inflammatory, antidiarrhoeal and anticonvulsant	Phenolics, flavonoids, saponins, terpenoids, glycosides, sterols, coumarins and quinones	[160]
*Strychnos henningsii* Gilg	Antioxidant, cytotoxic, antidiabetic, analgesic, anti‐inflammatory, antibacterial and antiplasmodial	Alkaloids, flavonoids, terpenes and phenols	[161]
*Strychnos innocua* Delile	Antimicrobial, antioxidant, antifungal, antiviral, anti‐inflammatory, antiprotozoal, hepatoprotective, angelic, antitumour and cytotoxic	Terpenoids, phenolics, saponins, flavonoids	[162]
*Strychnos spinosa* Lam.	Antimalaria, anti‐inflammatory, antioxidant cytotoxic	Alkaloids, terpenes, sterols, flavonoids, saponins	[163]
*Synaptolepis kirkii* Oliv.	Neurotrophic	Terpenoids	[164]
*Tamarindus indica* L.	Antibacterial, antifungal, antioxidant, antimicrobial, anticancer, antitubercular, antiviral, antidiabetic, anti‐inflammatory and cytotoxic	Phenols, flavonoids, saponins, tannins, alkaloids and steroids	[165]
*Terminalia sambesiaca* Engl. & Diels	Antioxidant	Phenols	[166]
*Uvaria acuminata* Oliv.	Antibacterial, antivenin, anti‐inflammatory, cytotoxic, anticancer, anticonvulsant, antimalarial and antianaemic	Flavonoids and xanthone	[167]
*Uvaria leptocladon* Oliv.	Anticonvulsant, antimicrobial, antioxidant, antivenom and anti‐inflammatory	Tannins, alkaloids, cardiac glycoside, flavonoids, phenols, quinones and saponins.	[168]
*Uvaria lucida* Bojer ex Benth.	Antidiabetic, anticancerous, anticonvulsant, antimicrobial, antioxidant, antiprotozoal, antivenom and anti‐inflammatory	Flavonoids	[167]
*Vachellia nilotica* (L.) P.J.H.Hurter & Mabb.	Antidiarrheal, astringent, anti‐inflammatory, analgesic, antipyretic, antioxidant, antihypertensive, antibacterial, antifungal, anticancer, antiplatelet, antiviral, anthelmintic and anti‐acetylcholinesterase	Quercetin, phenols, flavonoids, tannins, terpenoids and alkaloids	[169]
*Vangueria infausta* Burch.	Antifungal and antimalarial	Phenolics, tannins, coumarins, saponins, terpenoids, flavonoids and anthraquinones	[170]
*Waltheria indica* L.	Antimalarial, antifungal, antianaemic, anticonvulsants, sedative, anti‐inflammatory, antioxidant and antimicrobial	Steroids, alkaloids, terpenoids, flavonoids, tannins, saponins, anthraquinones and phenols	[171]
*Withania somnifera* (L.) Dunal	Antioxidant, antiepileptic, anti‐Alzheimer, cardio‐and hepato‐protective, and antiviral	Alkaloids, saponins, flavonoids, tannins, phenolics and steroids	[172, 173]
*Ximenia americana* L.	Antimicrobial, antifungal, anticancer, antineoplastic, antitrypanosomal, antirheumatic, antioxidant, analgesic	Flavonoids, steroids, tannins, alkaloids, phenolics, saponins, terpenoids and glycosides.	[174]
*Xymalos monospora* (Harv.) Baill.	Antioxidant	Phenols	[175]
*Zanha africana* (Radlk.) Exell	Antimycobacterial, antibacterial, antifungal, antiviral, antitrypanosomal, antidiabetic, anti‐inflammatory and cytotoxic	Anthocyanins, coumarins, saponins, steroids, tannins and triterpenoids	[176]
*Zanthoxylum capense* (Thunb.) Harv.	Antioxidant, cytotoxic and anti‐inflammatory	Alkaloids, coumarins, flavonoids, triterpenoids and phenols	[177]
*Zanthoxylum chalybeum* Engl.	Antidiabetic, antimalarial, antibacterial, antifungal and antiplasmodial	Alkaloids, coumarins, flavonoids, steroids, triterpenes, saponins and glycosides	[178]

### 3.8. Conservation Status

**FIGURE 4:**
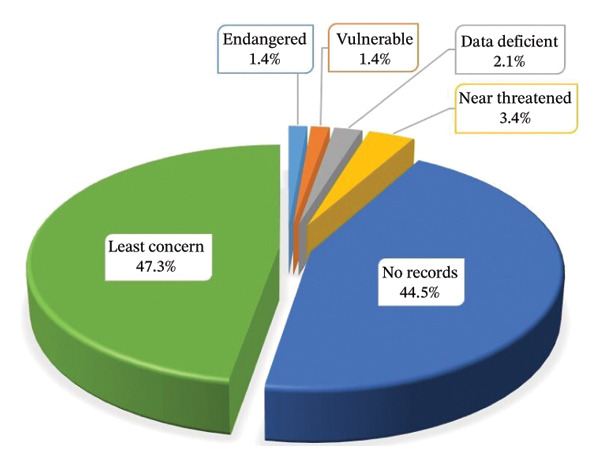
Percentage conservation status of the documented medicinal plants.

The conservation status of the documented MPs was verified using the International Union for Conservation of Nature (IUCN) Red List of Threatened Species website (IUCN 2022). Among the recorded MPs in this review, 47.3% (69 species) were listed as least concern, 3.4% (5 species) were near threatened, 2.1% (3 species) were data deficient, 1.4% (2 species) were vulnerable, 1.4% (2 species) were endangered, and 44.5% (65 species) had no records in the IUCN database (Figure 4). These findings suggest that most documented MPs in the country maintain stable populations. It is imperative to implement conservation efforts for species classified as near threatened, vulnerable or endangered. Furthermore, additional research is needed on the conservation status of MPs that remain unclassified in the database.

### 3.9. Need for Conserving Ethnomedicinal Knowledge

The global consumption of MPs is increasingly alarming, posing a significant threat to their existence [187]. An estimated 15,000 MPs are at risk due to heightened demand and the impacts of climate change [188]. Therefore, conserving MPs is critical, as is preserving indigenous culture and ethnomedicinal knowledge. This tradition of orally transmitting plant‐based medicine may be lost without adequate documentation [189]. Recently, younger generations have shown a growing disinterest in their heritage, frequently migrating for educational and employment opportunities, resulting in a neglect of traditional herbal health systems in favour of contemporary lifestyles. Shifts in lifestyles and socioeconomic conditions impede their connection to traditional knowledge [52, 190–192]. Due to their perceived convenience, young individuals residing in urban areas often opt for modern medications over herbal remedies. Moreover, agricultural expansion in rural locales threatens MPs, as numerous wild environments are cleared for farmland [193]. Consequently, thorough documentation is essential for conserving indigenous knowledge of traditional medicines. This review aspired to furnish foundational data for pharmacological studies seeking to develop contemporary medications for PAs.

## 4. Conclusion

Since antiquity, traditional medicinal practices have been employed to address a broad spectrum of human ailments, including those affecting paediatric populations. In this review, 146 MPs are documented for their use in the management of PAs. The majority of these species belong to the family Fabaceae. Shrubs constitute the most frequently employed life form, while leaves represent the predominant plant parts used in remedy preparation. Most plant materials are harvested from wild habitats, with a considerable proportion demonstrating pharmacological efficacy. The findings underscore the heightened vulnerability of children to diverse health challenges, thereby emphasising the importance of systematically documenting MPs used in paediatric care. Such documentation provides a foundation for future ethnopharmacological investigations, including chemical characterisation, toxicological assessment and context‐specific validation. Moreover, the results highlight the necessity of mechanistic studies and comprehensive safety profiling, while supporting the identification of novel drug leads informed by indigenous knowledge. Beyond biomedical implications, this review contributes to cultural conservation, the safeguarding of natural resources and the promotion of sustainable practices at both national and global levels.

## Author Contributions

David Sylvester Kacholi and Neema Gideon Mogha designed the study, analysed data, drafted the manuscript and approved the final submission.

## Funding

No funding was received for this research.

## Conflicts of Interest

The authors declare no conflicts of interest.

## Data Availability

The information supporting the findings of this study can be obtained from the corresponding author upon request.
